# Seropositivity to dengue and associated risk factors among non-malarias acute febrile patients in Arba Minch districts, southern Ethiopia

**DOI:** 10.1186/s12879-020-05370-3

**Published:** 2020-08-31

**Authors:** Daniel Eshetu, Techalew Shimelis, Eshetu Nigussie, Girma Shumie, Wakwoya Chali, Biruck Yeshitela, Abraham Assefa, Endalamaw Gadisa

**Affiliations:** 1 Department of Microbiology, Yirgalem Medical College, Yirgalem, Ethiopia; 2 Malaria-NTD Research Directorate, Armauer Hansen Research Institute, Addis Ababa, Ethiopia; 3 School of Medical Laboratory Science, Hawassa University, Hawassa, Ethiopia; 4 Department of Medical Laboratory Science, Madda Walabu University, Bale Goba, Ethiopia

**Keywords:** Febrile illness, Dengue virus, Seroprevalence, Immunofluorescence

## Abstract

**Background:**

Dengue fever is an arthropod vector-borne disease transmitted to humans by infected Aedes mosquitoes. Ethiopia has a favorable ecology for arthropods and report high burden of acute febrile illnesses. However, the contribution of arboviral infections to the burden of acute febrile illnesses is barely known. In this study the seropositivity to dengue virus infection and associated risk factors were assessed in Arba Minch districts, southern Ethiopia.

**Methods:**

An institution based cross-sectional study was conducted in a consecutive group of 529 acute febrile patients between May to August 2016. Socio-demographic data, residence place and clinical signs and symptoms were collected using structured questionnaires. Sera were tested for anti-dengue IgG and IgM using Euroimmune indirect immunofluorescent assay. Data analysis was done using SPSS V-20 (IBM Corp, 2012). *P*-value < 0.05 was taken as statistically significant.

**Result:**

Seropositivity was 25.1% (133/529) and 8.1% (43/529) for anti- IgG and IgM respectively.

**Conclusion:**

The high IgM prevalence detected indicate the probability of active transmission with a potential of public health significance that calls for a proactive follow up of the communities in the study area to forecast and avert the risk.

## Background

Dengue fever is an infectious disease and endemic in many countries of the world, is a (re) emerging infection [1, 2]. The past decades, witnessed a sharp increase in incidence, with estimated 390 million infection and 500, 000 hospitalizations related with the potentially life threating severe form per year. About 20–25, 000, mainly in children, dengue virus (DENV) infection associated deaths occur each year [3].

The recorded history of DENV in Africa is traced as far back as 1926; having caused an epidemic in Durban, South Africa [4]. Like the global trend, in Africa epidemics of dengue increased drastically since 1980 [5]. Outbreaks were reported in more countries in Africa including Cape Verde, Cote d’Ivoire, Gabon, Senegal, Djibouti, Kenya and Sudan between 2009 and 2012 [6]. Most recently in 2013, Africa recorded DENV outbreaks in Angola, Kenya, Seychelles and Tanzania [7]. Since the first outbreak in Ethiopia (2013/14) that occurred in Dire Dawa town, areas with similar ecological features, Ada’ar in Afar (2014) regional state and Gode town in Somali (2014 and 2015) regional state had outbreaks [8]. Despite, the increased threat and repeated outbreaks in wide geographical settings, the epidemiology and public health significance of DENV is barely documented in Ethiopia.

DENV infection induces only partial immunity which confers transient protection against subsequent infection by other serotypes [9]. On the basis of severity, the disease is grouped into probable dengue and severe dengue [10]. Probable dengue patient is, a patient either lived in endemic area or had history of travel to endemic areas, such patient may have symptoms like fever, rash, headache, persistent vomiting, mucosal bleed, abdominal pain/tenderness, restlessness, liver enlargement and increased in hematocrit value with decreased in platelet count [11]. In Severe dengue, the patient may have symptoms like severe plasma leakage, fluid accumulation (ascites), respiratory distress; severe bleeding and organ involvement (like liver, CNS and heart) leading to dengue shock syndrome (DSS) and DHF [12] with a mortality rate up to 26% [13].

The laboratory methods for the diagnosis of DENV infection include virus isolation, and detection of viral Nucleic acid, Antigen and anti-DENV antibodies (serology). The serologic detection of IgM and IgG antibodies by ELISA (Enzyme-linked immunosorbent assay) and IFA (Indirect immunofluorescence assay) platforms [14].

Despite the high burden of acute febrile illness in southern Ethiopia (SNNPR, Southern Nations Nationalities Peoples Regional state Health Bureau report 2014/150) and outbreaks in other parts of the country, the impact of DENV infection remain unnoticed. Thus, this study aimed to assess the prevalence of the exposure and its associated risk factors among non-malaria acute febrile patients in Arba Minch districts, SNNPR, southern Ethiopia.

## Methods

Consecutive self-reported volunteers at the outpatient departments of selected health centers were recruited from May to August 2016 in Arbaminch district. The district is located in the southern part of Ethiopia, with climatic condition of semi-desert and means annual rain fall 1300.5 mm. Three selected health centers (Lante Health Center, Shele Health Center and Birbir Health Center) were included in the study. Those sites were selected based on reports of higher rate of febrile illness (Personal communication with Health Bureau of SNNPR). Five hundred twenty-nine non-malaria acute febrile patients, 37.5 °C axillary temperature at initial evaluation and less than 7 days of onset of symptoms, at the specified health facilities were enrolled.

Structured questionnaires were used to capture data on clinical signs, symptoms and socio-demographic related risk factors. Five ml of venous blood was collected aseptically from each study participant and the serum sample was separated into multiple Nunc tubes for each patient then transported using liquid nitrogen and stored in deep freezers (− 80 °C) until screened for anti-dengue IgG and IgM using IIFT (Indirect Immune Fluorescent Test). The test was carried out according to the directions of the manufacturers.

Data collected with questionnaires and laboratory investigations were double entered in REDCap data software to control error and analyzed using SPSS version 20. Study findings were explained in words and tables. In Bivariate analysis, variables that were found to have association with the outcome variable at *P*-value of 0.25 were entered into multivariable logistic regression to assess the relationship between socio-demographic and other relevant variables with dengue virus sero-status. Associations between dependent and independent variables were assessed and its strength was described using odds ratios and 95% confidence intervals. P-value less than 0.05 were interpreted as statistically significant.

## Results

Of the 529 acute febrile cases, participants within the age group 15–30 years accounted 47.45%, followed by age group between 31 and 45 years 19.86%. Of the study participants, 39.9% had only primary school level education, 29.0% had secondary and higher grade completed and 31.1% had no formal education. Most study participants (86.8%) were rural residents (Table 1). The seroprevalence of dengue was 25.1% (133/529) for IgG and 8.1% (42/529) for IgM (Table 1).

**Table 1:** The prevalence of exposure Dengue virus (*N* = 529) by socio-demography and residence place in Arba Minch districts southern Ethiopia, 2016

Variables	Total (%)	IgG Test n (%)		IgM Test n (%)		COR (95% CI)	
		IgG + Ve	IgG -Ve	IgM + Ve	IgM -Ve	IgG Test	IgM Test
**Sex**							
Male	226(42.7)	61 (11.5)	165 (31.2)	16 (3.2)	210 (39.7)	1	1
Female	303 (57.3)	72 (13.6)	231 (43.7)	26 (4.9)	277 (52.4)	0.8 (0.6–1.3)	1.2 (0.6–2.4)
**Age group**							
< 15 yrs	80 (15.13)	25 (4.7)	55 (10.4)	9 (1.7)	71 (13.4)	1	1
15–30	251 (47.5)	61 (11.5)	190 (35.9)	21 (4.0)	230 (43.5)	0.3 (0.2–1.1)	0.3 (0.3–1.9)
31–45	105 (19.9)	28 (5.3)	77 (14.6)	8 (1.5)	97 (18.4)	0.3 (0.4–1.7)	0.4 (0.4–1.8)
> 45	93 (17.6)	19 (3.6)	74 (14.0)	4 (0.8)	89 (16.8)	1.6 (0.8–3.1)	1.4 (0.7–2.9)
**Residence**							
Rural	459 (86.8)	113 (21.4)	346 (65.4)	34 (6.4)	425 (80.4)	0.8 (0.5–1.4)	0.6 (0.3–1.4)
Urban	70 (13.2)	20 (3.8)	50 (9.4)	8 (1.5)	62 (11.7)	1	1

The seropositivity of resent mosquito bites 25.2. % and 6.6, habit of staying outside at the night 19.5 and 5.7%, use of bed-net at previous night17.8 and 6.1%, and presence of stagnant water in the village 8.1% and 2,7% IgG and IgM respectively. However, there was no statistically significant association between those associated factors and dengue virus infection (Table 2).

**Table 2:** Dengue virus infection in relation to risk factors in febrile patients (*N* = 529) in Arba Minch districts in southern Ethiopia, 2016

Variables	Total N (%)	IgG test n (%)		IgM test n (%)		COR (95% CI)	
		IgG + Ve	IgG -Ve	IgM + Ve	IgM -Ve	IgG test	IgM test
**Use of bed-net** **at previous night**							
Yes	360 (68.1)	94 (17.8)	266 (50.3)	32 (6.1)	328 (62.0)	0.9 (0.6–1.3)	1.6 (0.7–3.2)
No	169 (31.9)	39 (7.4)	130 (24.5)	10 (1.9)	159 (30.1)	1	1
**Presence of stagnant** **water in the village**							
Yes	172 (32.5)	43 (8.1)	129 (24.4)	14 (2.7)	158 (29.9)	1	1
No	357 (67.5)	90 (17.0)	267 (50.5)	28 (5.3)	329 (62.2)	1.0 (0.7–1.5)	1.0 (0.5–2.0)
**Habit of staying outside at the night**							
Yes	414 (78.3)	103 (19.5)	311 (58.8)	30 (5.7)	384 (72.6)	1.1 (0.7–1.7)	0.7 (0.3–1.4)
No	115 (21.7)	30 (5.7)	85 (16.0)	12 (2.3)	103 (19.5)	1	1
**Resent mosquito bite**							
Yes	246 (46.5)	62 (25.2)	184 (21.3)	35 (6.6)	211 (39.9)	1.0 (0.6–1.7)	1.0 (0.4–2.2)
No	283 (53.5)	71 (25.1)	212 (28.4)	7 (1.3)	276 (52.2)	1	1

The exposure of dengue virus infection in relation to different clinical factors is summarized in (Table 3). Majority 21.2% and 2, 7% of the study participants had constitutional symptoms, followed by high body temperature 11.9 and 3.4% then Headache 8.1 and 2.1% for IgG and IgM respectively. Other clinical features that the study participants experienced include: sore throat 7.2 and 4.0%, crepitation 3.6 and 1.5%, flank pain 1.9 and 0.8%, flank pain 1.9 and 0.6%, abnormal heart sound 1.5 and 0.6%, rash 1.1 and 0.4%, blurred vision 0.8 and 0.4%, neck stiffness 0.6% and 0,2%, musculoskeletal tenderness 0.6% and 0,2%, hearing loss 0.2 and 0% (Table 3).

**Table 3:** Dengue virus infection in relation to clinical features in febrile patient in Arbaminch Districts in southern Ethiopia, 2016

Variables	Total N(%)	IgG test n (%)		IgM test n (%)		COR (95% CI)	
		IgG + Ve	IgG -Ve	IgM + Ve	IgM -Ve	IgG test	IgM test
**Head signs (scalp lesion)**							
Yes	17 (3.2)	4 (0.8)	13 (2.4)	0 (0)	17 (3.2)	1	1
No	512 (96.8)	129 (24.4)	383 (72.4)	42 (8.0)	470 (88.9)	1.1 (0.4–3.4)	0.92 (2.9)
**Neck signs (neck stiffness)**							
Yes	20 (3.8)	3 (0.6)	17 (3.2)	1 (0.2)	19 (3.6)	1	1
No	509 (96.2)	130 (24.6)	379 (71.6)	41 (7.8)	468 (88.5)	0.9 (0.2–3.4)	1.2 (0.2–9.3)
**Eye symptoms (blurred vision)**							
Yes	61 (11.5)	4 (0.8)	57 (10.7)	2 (0.4)	59 (11.2)	1	1
No	468 (88.5)	129 (24.4)	339 (64.1)	40 (7.6)	428 (80.9)	1.5 (0.5–4.6)	0.5 (0.1–4.1)
**Ear symptoms (hearing loss)**							
Yes	9 (1.7)	1 (0.2)	8 (1.5)	0 (0)	9 (1.7)	1	1
No	520 (98.3)	132 (25.0)	388 (73.3)	42 (7.9)	478 (90.4)	0.2 (0.0–1.9) *	1.0 (0.1–8.2)*
**Mouth and Throat (sore throat)**							
Yes	59 (11.2)	38 (7.2)	21 (4.0)	14 (2.7)	45 (8.5)	1	1
No	470 (88.8)	95 (18.0)	375 (70.8)	28 (5.3)	442 (83.6)	0.9 (0.5–1.3)	0.7 (0.4–1.3)
**Nose sign (nose bleeding)**							
Yes	25 (4.7)	9 (1.7)	16 (3.0)	3 (0.6)	22 (4.2)	1	1
No	504 (95.3)	124 (23.4)	380 (71.9)	39 (7.4)	465 (87.9)	1.2 (0.5–2.6)	2.8 (0.4–20.9)
**Chest exam signs (crepitation)**							
Yes	29 (5.5)	19 (3.6)	10 (1.9)	8 (1.5)	21 (4.0)	1	1
No	500 (94.5)	114 (21.6)	386 (72.9)	34 (6.4)	466 (88.1)	0.7 (0.1–6.7)	0.6 (0.3–1.4)
**Cardiovascular signs (abnormal heart sound)**							
Yes	30 (5.7)	8 (1.5)	22 (4.2)	2 (0.4)	28 (5.3)	1	1
No	499 (94.3)	125 (23.6)	374 (70.7)	40 (7.6)	459 (86.8)	1.1 (0.5–2.5)	1.1 (0.2–1.1)
**Abdominal signs (enlarged liver)**							
Yes	43 (8.1)	10 (1.9)	33 (6.2)	3 (0.6)	40 (7.56)	1	1
No	486 (91.9)	123 (23.3)	363 (68.6)	39 (7.4)	447 (84.5)	1.3 (0.6–2.7)	0.8 (0.2–2.6)
**Urination symptoms (flank pain)**							
Yes	43 (8.1)	10 (1.9)	33 (6.2)	4 (0.8)	39 (7.4)	1	1
No	486 (91.9)	123 (23.3)	363 (68.6)	38 (7.2)	448 (84.7)	1.3 (0.6–2.7)	0.6 (0.2–1.9)
**Musculoskeletal Signs (tenderness)**							
Yes	15 (2.8)	3 (0.6)	12 (2.2)	1 (0.2)	14 (2.7)	1	1
No	514 (97.2)	130 (24.6)	384 (72.6)	41 (7.8)	473 (89.4)	0.7 (0.2–2.7)	1.2 (0.2–9.5)
**Dermatological Signs (rash)**							
Yes	16 (3.0)	6 (1.1)	10 (1.9)	2 (0.4)	14 (2.7)	1	1
No	513 (97.0)	127 (24.0)	386 (73.0)	40 (7.6)	473 (89.4)	1.8 (0.7–5.1)	0.6 (0.1–2.7)
**High body Temperature**							
37.5–38	284 (53.7)	63 (11.9)	221 (41.8)	18 (3.4)	266 (50.3)	0.7 (0.5–1.1) *	1.6 (0.9–3.0) *
> 38	245 (46.3)	70 (13.2)	175 (33.1)	24 (4.5)	221 (41.8)	1	1
**Headache**							
Yes	441 (83.4)	109 (8.2)	332 (1.4)	11 (2.1)	430 (81.3)	0.9 (0.5–1.5)	0.8 (0.3–2.0)
No	88 (16.6)	24 (1.8)	64 (14.8)	31 (5.9)	57 (10.8)	1	1
**Shortness of breath**							
Yes	24 (4.5)	13 (2.5)	11 (2.0)	3 (0.6)	21 (4.0)	1	1
No	505 (95.5)	120 (22.7)	385 (72.8)	39 (7.4)	466 (88.1)	1.0 (0.40–4.40)	1.0 (0.1–8.2)
**Vomiting**							
Yes	85 (16.1)	21 (4.0)	64 (12.1)	6 (1.1)	79 (1493)	1	1
No	444 (83.9)	112 (21.2)	332 (62.7)	36 (6.8)	408 (77.1)	1.0 (0.6–1.7)	1.2 (0.5–2.9)
**Diarrhea**							
Yes	45 (8.5)	8 (1.5)	37 (7.0)	2 (0.4)	43 (8.1)	1	1
No	484 (91.5)	125 (23.6)	359 (67.9)	40 (7.6)	444 (83.9)	0.6 (0.28–1.4)*	1.9 (0.5–8.3)*
**Constitutional symptoms**							
Yes	412 (77.9)	112 (21.2)	300 (56.7)	14 (2.7)	398 (75.2)	1.71 (1.0–2.9)**	1.4 (1.1–2.0)**
No	117 (22.1)	21 (4.0)	96 (18.1)	26 (4.9)	91 (17.2)	1	1

*COR* crude odds ratio; *CI* confidence interval; *1* Reference; * Candidate variable (*p* < 0.25); ** Significant association (*p* < 0.05)

In bivariate analysis, candidate variables like: Ear symptoms, Diarrhea, high body temperature and constitutional symptoms, also occupation like employee, student and age group between 31 and 45 years and age group > 45 years are selected. To avoid the possible confounding variables that found to have association with the dengue virus at *P*-value of 0.25 by bivariate were entered into multivariate logistic regression. Only constitutional symptoms remain significant (OR = 1.71; 95% CI 1.015–2.870; *p* < 0.044);of 112 who had constitutional symptoms and positive 10.0% had only acute fever, 7.5% had acute fever and fatigue, 19.4% had fever and loss of appetite, 31.3% had fever, fatigue and loss of appetite, and 1.7% had acute fever, loss of appetite and night sweeting.

## Discussion

The finding of this study shows, the overall seroposetivity of dengue virus exposure in this study was 25.1% for IgG and 8.1% for IgM. The distribution of dengue seropositivity rate among the study population in the 3 health facilities was comparable. This suggests that the risk of being infected by dengue is relatively homogenous within the populations from each health facility. Direct comparison between different findings may be difficult due to methodological difference, diagnostic tools employed and study population investigated. This finding is comparable to the rate (27.7%) that reported from a border state between Sudan and Republic of South Sudan [15]. The current result was also in line with the finding of a study conducted in Djibouti (21.8%) [16]. However, was lower than the reports from Dire Dawa, Ethiopia [17], in Nigeria [18], in Kenya [19], and in Brazil [19], which were 62.5, 67.7, 67.0 and 56%, respectively.

On the other hand, the lower rates of seropositivity compared to the current finding were reported in studies done in Kenya [20], in Cameroon [21], in north-west Zambia [22] and in eastern part of Sudan [23],which were 14.4, 12.5, 4.1 and 9.4%, respectively. The reason for this difference in prevalence between the previous studies and the current study may be due to various factors. The current study focused on the detection of IgG/IgM antibodies from acute febrile participants; however, the previous studies focused mainly on detection of IgG antibodies from healthy participants (survey on healthy individuals) or detection of IgG antibodies in dengue outbreak situations. Diagnostic tools also make difference as there is a considerable discrepancy between the performance of indirect ELISA and PRNT (Plague Reduction Neutralization test) [24].

Further, in line with the study result in Texas-Mexico [25], this study showed no association between dengue exposure and utilization of bed net, use of mosquito repellent, presence stagnant water near resident areas and tree in the compound, staying out side at night, or knowledge about the virus. However, in contrast to these findings, a study in Haiti suggested that utilization of bed net might protect people against dengue infection [26]. The lack of association in the current study may be because *Aedes aegyptics* commonly bites during day time [11], and therefore the use of bed net may not help provide a barrier between humans and the vector.

As a cause of febrile illness, dengue virus infection is characterized by clinical features including high fever, headache, severe myalgia, nausea and vomiting and frequently rash. However, the predominant clinical signs and symptoms of the infection may vary with populations in different geographical regions [27]. It was shown in a study conducted in Kenya that headache; muscle pain, joint pain and abdominal pain were found to be associated with increased rate of dengue virus infection. However, none of those variables were associated with the infection in the current study as was the case in a study done in rural parts of western Kenya [28].

The limitations of this study were its cross-sectional nature and the use of serology (Euroimmune IIFT) test alone to determine the seroposetivity of Dengue virus. Therefore, the findings may not be generalized to the general population in the study area and even if the manufacturer claimed high specify of the test to our knowledge no comparative studies confirming that and thus probability of cross reacting with other arboviral infection could not be ruled out.

## Conclusion

The high IgM prevalence detected indicate the probability of active transmission with a potential of public health significance that calls for a proactive follow up of the communities in the study area to forecast and avert the risk.

## Data Availability

The dataset used for this article is available upon request to Armauer Hansen Research Institute. Written authorization from AHRI/ALERT ethics committee (P.O. Box 1005, Addis Ababa) and Endalamaw Gadisa (Dr) email: endalamaw.gadisa@ahri.gov.et.

## Abbreviations

AHRI: Armauer Hanen Research Institute
DENV: Dengue virus
DF: Dengue Fever
OPD: Outpatient department
ELISA: Enzyme linked immune sorbent assay
RT-PCR: Reverse transcriptase Polymerase chain reaction
IIFA: Indirect immunofluorescence assay
IgG: Immunoglobulin G
IgM: Immunoglobulin M
AFI: Acute febrile illness
PRNT: Plaque reduction neutralization test
